# The Midgut Microbiota of Colombian *Aedes aegypti* Populations with Different Levels of Resistance to the Insecticide Lambda-cyhalothrin

**DOI:** 10.3390/insects11090584

**Published:** 2020-09-01

**Authors:** Andrea Arévalo-Cortés, Ana M. Mejia-Jaramillo, Yurany Granada, Heather Coatsworth, Carl Lowenberger, Omar Triana-Chavez

**Affiliations:** 1 Group Biología y Control de Enfermedades Infecciosas, Universidad de Antioquia, Calle 70 No. 52-21, Medellín 050010, Colombia; aarevalocortes@gmail.com (A.A.-C.); maria.mejia3@udea.edu.co (A.M.M.-J.); eresbey2@gmail.com (Y.G.); 2 Centre for Cell Biology, Development, and Disease, Department of Biological Sciences, Simon Fraser University, 8888 University Drive, Burnaby, BC V5A 1S6, Canada; hcoatswo@sfu.ca (H.C.); clowenbe@sfu.ca (C.L.)

**Keywords:** *Aedes aegypti*, microbiome, lambda-cyhalothrin, insecticide resistance

## Abstract

**Simple Summary:**

*Aedes aegypti* is a mosquito capable of transmitting many viral diseases such as dengue, Zika, and chikungunya. Since no effective treatments are available for these viruses, eliminating the mosquito with insecticides is vital to combat these diseases. However, the mosquito can generate resistance to the insecticide by changing its genes or its physiology. It has been recognized that the type of bacteria that live inside the mosquito’s gut can contribute to this resistance. In this study, we evaluated *Ae. aegypti* mosquitoes from six locations in Colombia to determine if they are resistant to lambda-cyhalothrin insecticide, and we analyze their gut microbiota. We observed resistance in five of the six areas. We compared the gut microbiota from susceptible and resistant mosquitoes and found specific bacteria in resistant mosquitoes that may play a role in insecticide resistance. Overall, our findings contribute to the understanding of insecticide resistance in *Ae. aegypti* that will generate alternatives for interventions to control this mosquito in Colombia.

**Abstract:**

Insecticide resistance in *Aedes aegypti* populations is a problem that hinders vector control and dengue prevention programs. In this study, we determined the susceptibility of *Ae. aegypti* populations from six Colombian regions to the pyrethroid lambda-cyhalothrin and evaluated the presence of the V1016I mutation in the sodium channel gene, which has been broadly involved in the resistance to this insecticide. The diversity of the gut microbiota of these mosquito populations was also analyzed. Only mosquitoes from Bello were susceptible to lambda-cyhalothrin and presented a lower allelic frequency of the V1016I mutation. Remarkably, there was not an important change in allelic frequencies among populations with different resistance ratios, indicating that other factors or mechanisms contributed to the resistant phenotype. Treatment of mosquitoes with antibiotics led us to hypothesize that the intestinal microbiota could contribute to the resistance to lambda-cyhalothrin. Beta diversity analysis showed significant differences in the species of bacteria present between susceptible and resistant populations. We identified 14 OTUs of bacteria that were unique in resistant mosquitoes. We propose that *kdr* mutations are important in the development of resistance to lambda-cyhalothrin at low insecticide concentrations but insect symbionts could play an essential role in the metabolization of pyrethroid insecticides at higher concentrations, contributing to the resistant phenotype in *Ae. aegypti*.

## 1. Introduction

The mosquito *Aedes aegypti* is the biological vector of viruses such as dengue, Zika, and chikungunya. Dengue affects 390 million people around the world [1]. For Zika, over 2 billion people live in regions conducive to the transmission and in 2016 the infection spread rapidly in the Americas, where approximately 4 million infections were predicted, and major outbreaks were reported in Brazil [2,3]. Chikungunya originated in Africa and has caused epidemics in Asia, the Indian subcontinent, Europe, the Americas, and the Pacific Islands, where millions of people have been infected [4]. With no suitable vaccines currently available, vector control through the use of chemical insecticides is the primary measure existing to prevent these diseases. However, insecticide resistance is a latent problem for the control of mosquito populations. A plethora of previous studies on the molecular mechanisms of insecticide resistance has focused on metabolic resistance and point mutations in individual genes that render the insect resistant. For instance, target site insensitivity resistance (knockdown resistance (*kdr*) mutations, frequently caused by nonsynonymous mutations in the voltage-gated sodium channel transmembrane protein) and metabolic detoxification, have been involved in the loss of sensitivity to insecticides such as the pyrethroid lambda-cyhalothrin [5,6,7,8,9,10]. However, there are factors beyond these features that could contribute to the resistant/susceptible phenotype, including microorganisms that potentiate the degradation of xenobiotics [11,12].

Bacteria of the genus *Burkholderia* have been linked to insecticide resistance against the organophosphate pesticide fenitrothion in *Riptortus pedestris* (stinkbugs) [13,14]. Greater levels of Lactobacillales, and the much scarcer taxa Pseudomonadales, and Xanthomonadales are found in strains of *Plutella xylostella* (diamondback moth) resistant to chlorpyrifos (organophosphate) and fipronil (phenylpyrazole) [15]. More recently, an association between *Bacillus cereus* and *Bacillus thuringiensis* and metabolization of organophosphates, pyrethroids, fipronil, and DDT was identified in fenitrothion-resistant *Anopheles albimanus* [12]. Likewise, bacteria such as *Pseudomonas stutzeri*, *Pseudomonas oleovorans*, *Arthrobacter nicotinovorans*, *Enterococcus mundtii*, and *Klebsiella sp.* have been reported to degrade insecticides such as the pyrethroids lambda-cyhalothrin and deltamethrin [11,16,17]. Symbiotic bacteria, involved in the degradation of xenobiotics, have been reported principally in agricultural pests, and are used in bioremediation processes [11,16,18,19].

In Colombia, insecticide resistance has been progressive, rendering control of *Ae. aegypti* difficult in some regions of the country, even though control strategies use a rotation of different types of insecticides [5,6,7,8,9,10,20,21]. The organophosphate temephos has been used for larval control in Colombia since 1970, and pyrethroids have been used as adulticides since 1990 [5,9], and the resistance against both types of insecticides has been reported in *Ae. aegypti* [5,6,7,8,20,21]. The V1016I *kdr* mutation has been related with resistance to pyrethroids, including lambda-cyhalothrin, in several parts of the world [8,9,10,22,23,24,25,26,27,28,29]. Although 11 *kdr* mutations have been reported worldwide in *Ae. aegypti*, only three have been observed in Colombia, and of them, only the V1016I mutation has been involved in the lambda-cyhalothrin resistance. This mutation was described in the populations of *Ae. aegypti* from the Caribbean region of Colombia [9,23], several municipalities of Valle del Cauca and cities such as Giron, Medellín, Villavicencio, and Riohacha [8,10].

In this study, we analyzed the V1016I mutation in the sodium channel gene in mosquitoes from six Colombian regions with different lambda-cyhalothrin susceptibility profiles. The results indicated that the resistance is only partially explained through the *kdr* mechanism. Therefore, we hypothesized that midgut microbiota could also contribute to insecticide resistance in *Ae. aegypti* populations in Colombia. We analyzed the midgut microbiota from adult female *Ae. aegypti* collected in these different locations. Although the bacterial core was the same for all regions, we found some differences in bacterial populations that might contribute to the resistant phenotype.

## 2. Materials and Methods

### 2.1. Mosquito Collections

**Figure 1:**
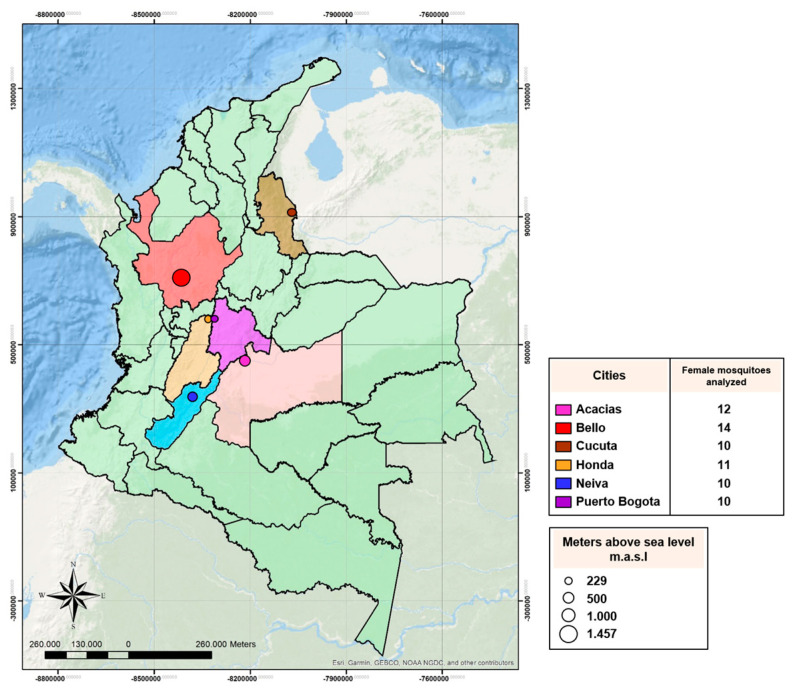
Map of Colombia showing cities where samples of *Aedes aegypti* mosquitoes were collected. The circle size denotes the altitude of each city. The number of female mosquitoes analyzed is indicated in the rectangle.

Adults and immature stages of *Ae. aegypti* were collected between 2016 and 2017 in six Colombian cities with the assistance of staff involved in vector-borne disease programs from each municipality. The mosquitoes were collected using sweep nets, in 10 to 20 randomized houses from four neighborhoods. The location and metadata associated with the mosquito samples analyzed in this study are shown in Figure 1 and Table S1. Female mosquitoes were immediately placed in individual tubes and maintained on ice until subsequent taxonomic identification was done. F0 field females destined for analysis of bacterial diversity were stored at −80 °C until DNA extraction was performed for metagenome 16S sequencing. Immature stages were reared to adults under controlled conditions: temperature (28 ± 1 °C), relative humidity (80 ± 5%), and photoperiod (12 h light:12 h dark). F0 adults were used for the analysis of the V1016I mutation, F1 larvae to determine the insecticide resistance profile with bioassays, and F6 adult females for the insecticide bioassays plus antibiotics.

For the insecticide bioassays with axenic females, larvae were reared under selection conditions with lambda-cyhalothrin. F2 and F3 larvae were exposed to 0.015 ppm of lambda-cyhalothrin (lethal concentration 50, LC50). The dose was increased in F4 larvae to 0.05 ppm (LC90). This dose was maintained until obtaining F6 adult females, with whom we worked.

### 2.2. Determination of Lambda-Cyhalothrin Resistance Profile of Larvae

Mosquitoes from each location were screened for susceptibility to the insecticide lambda-cyhalothrin (99.7%) purchased from Sigma-Aldrich (USA), which is used commonly in the Colombian public health programs to reduce mosquito populations. Although pyrethroids are not used for larval treatment, we tested them against *Ae. aegypti* to obtain information on the larval resistance status that may reflect the adult resistance status since the target of pyrethroids is a constitutively expressed gene. Sixty F1 larvae from the third or fourth instar were exposed to one of six concentrations of lambda-cyhalothrin (0.000468 ppm to 0.06 ppm) to determine larval mortality 24 h after exposure, following the standardized methods of the World Health Organization (WHO) [30]. The insecticide-susceptible Rockefeller strain was used as a control. Three biological and technical replicates were performed at each concentration as well as unexposed controls [8]. The lethal concentrations (LC50) and 95% confidence intervals (CIs) (*p* < 0.05) were calculated for each population. Concentration-mortality data were subjected to Probit analysis and 95% CIs, and resistance ratios (RRs) were calculated. A resistance ratio (RR) was obtained by dividing the LC50 of each population by the equivalent LC50 of the Rockefeller reference strain. The RR was interpreted as susceptible to insecticide if <5-fold, moderate insecticide resistance (5- to 10-fold), and high insecticide resistance (>10-fold) [30]. *p*-values < 0.05 were considered statistically significant.

### 2.3. Allele-Specific PCR (AS-PCR) for the kdr Mutation V1016I

Analysis of the V1016I mutation was carried out on the F0 generation of each population. Mosquitoes were cold sacrificed and stored at −20 °C in 1.5 mL vials with molecular grade alcohol, for subsequent DNA extraction. Extraction was carried out using the Grinding Buffer method [31] and the ZR Tissue and Insect DNA MiniPrep^TM^ kit (ZYMO RESEARCH, Irvine, CA, USA; Catalog No. D6016).

For allele-specific PCR, we used primers previously developed to mutation 1016 (G3046A), which were previously reported by Li et al., 2015: PM2_Ext_1016F GCCACCGTAGTGATAGGAAATC; PM2_Ext_1016R CGGGTTAAGTTTCGTTTAGTAGC [32], and Granada et al., 2018: PM2_F_1016Wt GTTTCCCACTCGCACAGGT; PM2_F_1016Mut GTTTCCCACTCGCACAGA [8]. The AS-PCR was performed in a final volume of 25 µL which contained 1.5 µL MgCl_2_ [25 mM], 2.5 µL of Thermo buffer [10×], 1.25 µL of each of the primers [10 mM], 1.25 µL dNTPs [10 mM], 0.25 µL of Taq Thermo, and 2 µL of DNA. Thermal profile conditions were 94 °C for 30 s, followed by 35 cycles of 94 °C for 30 s, 60 °C for 1 min, 72 °C for 1 min, and finally an extension of 72 °C for 7 min. The PCR product size was separated by electrophoresis on 1.5% agarose gel in a TBE buffer, at 100 V for 60 min, stained with ethidium bromide, and visualized under UV light on a photo-documentator (BIO-RAD^®^ Laboratories, Hercules, CA, USA). The Rockefeller strain of *Ae. aegypti* was used as a reference for the wild-type allele (1016V) of the sodium channel gene. The size of the PCR products for the detection of wild and mutated alleles in each of the mosquitoes was 348 bp (V1016I).

The allele frequencies were determined by comparing the number of each of the alleles (wild and mutated) with the total number of alleles in the population, using the Microsoft Excel program. To assess whether there was a relationship between the frequency of the mutated allele at position V1016I of *Ae. aegypti* and resistance ratio (RR_50_) to lambda-cyhalothrin, an analysis of the Pearson correlation coefficient (*r*^2^) (*p* < 0.05) was done. This analysis was performed using GraphPad Prism Software (version 5.1 for Windows, GraphPad Software, La Jolla, CA, USA, www.graphpad.com).

### 2.4. Generation of Axenic Adult Mosquitoes

To identify the role of bacteria in resistance, pupae from the Acacias mosquito population, which was the most lambda-cyhalothrin resistant population, were kept under sterile conditions, and emerging females were fed with sterile 10% sucrose solution containing 50 U/mL penicillin and 50 µg/mL streptomycin (P/S) for three days, following the methodology proposed by Ramirez et al. [33]. These females were then placed in groups of 20 in 250 mL sterile bottles impregnated with 10 ppm lambda-cyhalothrin for 30 min. Mortality was recorded every 5 min following the CDC test criteria [20]. For this analysis, two-way ANOVA and Bonferroni tests were applied using GraphPad Prism Software (version 5.1 for Windows, GraphPad Software, La Jolla, CA, USA, www.graphpad.com).

### 2.5. Preparation of Genomic DNA, and Library for Metagenome 16S Sequencing

At least 10 adult female mosquitoes collected from each municipality (Figure 1) were morphologically identified as *Ae. aegypti*. These mosquitoes were surface-sterilized by dipping them in 70% ethanol for 5 min and then rinsed twice for 1 min in sterile Dulbecco’s phosphate-buffered saline (DPBS) solution. The midgut from each mosquito was dissected under sterile conditions in a drop of sterile DPBS, taking care not to contaminate it with any other tissues, and total DNA was extracted from each midgut with the ZymoBIOMICS^TM^ DNA Miniprep kit (ZYMO RESEARCH, Irvine, CA, USA; Catalog No. D4300). Dissections and extractions were performed in an aseptic environment to avoid contamination. Additionally, a mock sample spiked with the ZYMBIOMICS^TM^ Microbial Community Standard (ZYMO RESEARCH, Irvine, CA, USA; Catalog No. D6300), which contains samples from 10 well-known species of bacteria was used in all procedures as environmental contamination controls. The DNA was sent to Macrogen (Seoul, South Korea), where the libraries were prepared and sequenced. Briefly, DNA integrity was verified on a 2100 Bioanalyzer (Agilent Technologies, Santa Clara, CA, USA), and the prepared libraries were quantified using the Illumina qPCR Quantification Protocol Guide. The bacterial 16S V3–V4 region was sequenced for 68 DNA samples (67 from mosquito midguts, and the mock sample) on an Illumina Miseq platform using the bacterial and archaeal universal primers 16S_V3-341F: (5′-CCTACGGGNGGCWGCAG-3′) and 16S_V4-785R: (5′-GACTACHVGGGTATCTAATCC-3′).

### 2.6. Statistical Analyses

All data were analyzed using Quantitative Insights into Microbial Ecology 2 (QIIME2) (version 2019.1) [34]. Raw fastq files were trimmed to remove adapters and low-quality bases. The sequences were demultiplexed using DADA2 [35], and reads were truncated to avoid low-quality scores. The feature table, taxonomy data, and sample metadata were then imported into MicrobiomeAnalyst to be analyzed [36]. Samples were filtered for low abundance (10%), based on the mean abundance of operational taxonomic units (OTUs), and for variability using an inter-quantile range assessment. The data were normalized by rarefying to the minimum library size, and OTU abundances were transformed using the centered log-ratio.

### 2.7. Bacterial Diversity

The alpha diversity indices (observed OTUs, Chao1, and Shannon) among sample groups were tested using analysis of variance (ANOVA). To assess beta diversity differences, the groups were analyzed using principal coordinates analysis (PCoA), and non-metric multidimensional scaling (nMDS) plotting Bray-Curtis, Jensen-Shannon divergence, and Jaccard distance matrices, with Permutational Multivariate Analysis of Variance (PERMANOVA), as described previously [37]. Significance for the pair-wise comparisons was set to *p* value (i.e., FDR adjusted *p*-value < 0.05).

### 2.8. Taxonomic Annotation and Relative Abundance of Taxa

OTUs with 97% nucleotide sequence identity were assigned to known bacterial taxa based in the Greengenes database (v2018). Low abundance OTUs (≤10 counts) with 10% or lower prevalence in samples were removed. The taxa were grouped by abundance at different hierarchical levels (Phylum, Class, Order, Family, Genus, Species, and OTUs), and an analysis of microbiome composition (ANCOM) was used to test for differential abundance across multiple taxonomic levels using the MetagenomeSeq statistical pipeline [36]. A linear discriminant analysis effect size (LEfSe) was used to detect potential phenotype-specific bacterial markers. Our goal was to compare OTUs that were differentially abundant between lambda-cyhalothrin susceptible and resistant mosquitoes. All procedures were performed using MicrobiomeAnalyst [36].

### 2.9. Data Accessibility

All sequence reads obtained in this study have been deposited in the National Center for Biotechnology Information (NCBI) under the BioProject PRJNA547790.

## 3. Results

### 3.1. Mosquito Populations from Colombia Show Different Susceptibility Profiles to Lambda-Cyhalothrin

The RR_50_ of mosquitoes from the six Colombian cities, evaluated in third or fourth instar F1 larvae, ranged from 1.97 to 32-fold. The *Ae. aegypti* population from Bello was the only insecticide-susceptible population, with an RR of 1.97. Mosquitoes from Puerto Bogota presented a moderately resistant profile (RR = 9.14), and the rest of the populations were highly resistant (RR > 10.55) (Table 1).

### 3.2. Genotyping of kdr Mutation V1016I

**Figure 2:**
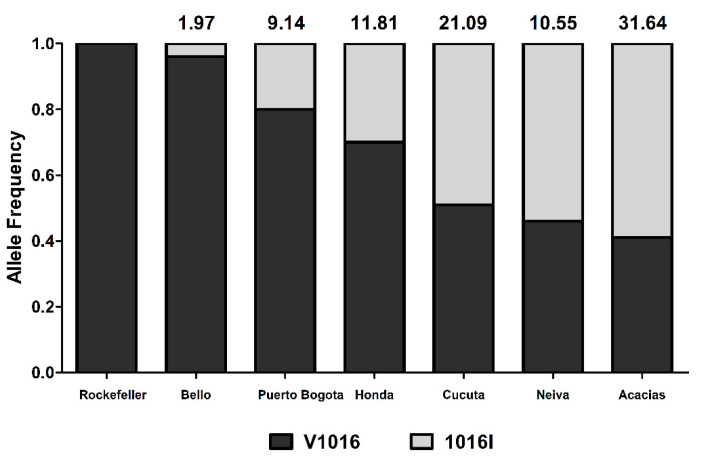
Comparison of allelic frequencies for the 1016I mutation in *Aedes aegypti* adults from six cities studied in Colombia. The resistance ratio (RR_50_) value for mosquitoes from each city is shown at the top of each column. The mutation in codon 1016 results in the replacement of valine (V) for isoleucine (I).

The frequencies of the 1016I mutant allele for F0 adults were 0.20, 0.30, 0.49, 0.54, and 0.59 for the resistant populations of Puerto Bogota, Honda, Cucuta, Neiva, and Acacias, respectively (Figure 2). We genotyped at least 50 adult mosquitos from each population. On the other hand, the Bello population, which was the only susceptible population of the six evaluated, had an allelic frequency of 0.04 (Figure 2). The Pearson correlation analysis indicated a poor association between allelic frequencies and resistance ratio (*r*^2^ = 0.6515, *p* > 0.05), suggesting that other mechanisms could be involved in the resistance to lambda-cyhalothrin.

### 3.3. Axenic Adult Mosquitoes are More Susceptible to Lambda-Cyhalothrin

**Figure 3:**
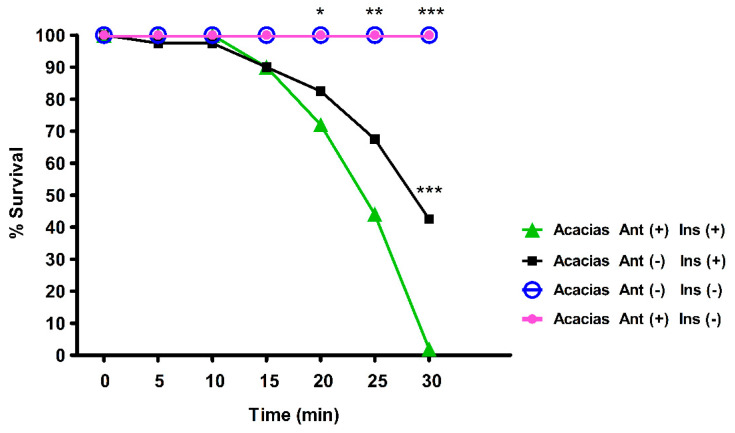
Mortality of lambda-cyhalothrin resistant mosquitoes treated with antibiotics (Ant). Females from the highly resistant Acacias strain F6 (selected by treatment with lambda-cyhalothrin) were treated (Ant +) or not (Ant −) with 50 U/mL penicillin/50 µg/mL streptomycin. The mortality after exposure to the insecticide lambda-cyhalothrin (Ins +) for 30 min was evaluated. Mosquitoes treated or not with antibiotics (Ant +/Ant −) but not exposed to insecticide (Ins −) were used as controls. Statistical analyses included two-way ANOVA and Bonferroni tests (*** *p* < 0.001; ** *p* < 0.01; * *p* < 0.05). There was a significant difference at 30 min between Ant (−) Ins (+) and Ant (+) Ins (+). These two treatments were significantly different from controls without insecticide at 20, 25, and 30 min.

To examine whether gut bacteria have a role in the resistance to lambda-cyhalothrin, we treated female mosquitoes with antibiotics for three days. These mosquitoes were subsequently exposed to lambda-cyhalothrin following the standardized methods of the of the CDC [20]. At 30 min post-exposition to lambda-cyhalothrin, 98% of the females treated with P/S died, while mortality was only 58% for females that had not been treated (*p* < 0.001, Figure 3). Controls treated with P/S but not with lambda-cyhalothrin survived the 30-min observation period. Females that were not treated with either lambda-cyhalothrin or antibiotics did not have any mortality (Figure 3). These results suggest that specific bacteria, or their metabolites, may contribute to the insecticide-resistant phenotype in Colombian *Ae. aegypti* populations.

### 3.4. Metagenome Analyses

A total of 4,312,781 reads were obtained from 68 samples (F0 female adults). The number of mean reads per sample was 69,263, and the mean read length was 298 bp (Figure S1). After data filtering, these reads were clustered at 97% sequence similarity into 8787 unique OTUs, of which 1204 contained phylum level information (Tables S2 and S3). Rarefaction curves indicated that sequencing coverage was sufficient (i.e., yielded a plateau) for all samples (Figures S2 and S3).

### 3.5. Alpha and Beta Diversity Analyses

**Figure 4:**
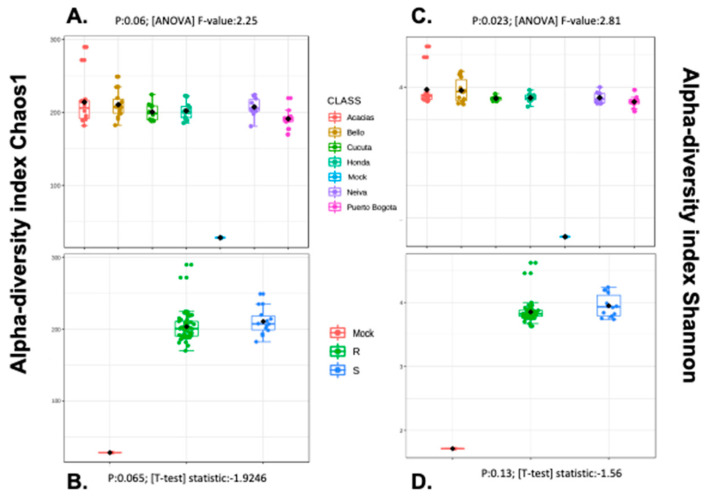
Alpha diversity (Chao1 and Shannon indices) among six Colombian *Aedes aegypti* populations. (**A**,**C**): Alpha diversity among geographical origins. (**B**,**D**): Alpha diversity between lambda-cyhalothrin resistant (R) and susceptible (S) mosquitoes. The mock sample corresponds to samples of known bacteria used as controls during DNA extraction procedures and sequencing.

Comparisons of the OTUs using several alpha diversity metrics (Chao1, Shannon) showed that there were no significant differences among geographical locations or resistance profiles (Figure 4, Table S4). Likewise, the number of observed OTUs was not significantly different among the samples (Table S4). However, there were exclusive OTUs in mosquitoes from each city (Table S5).

**Figure 5:**
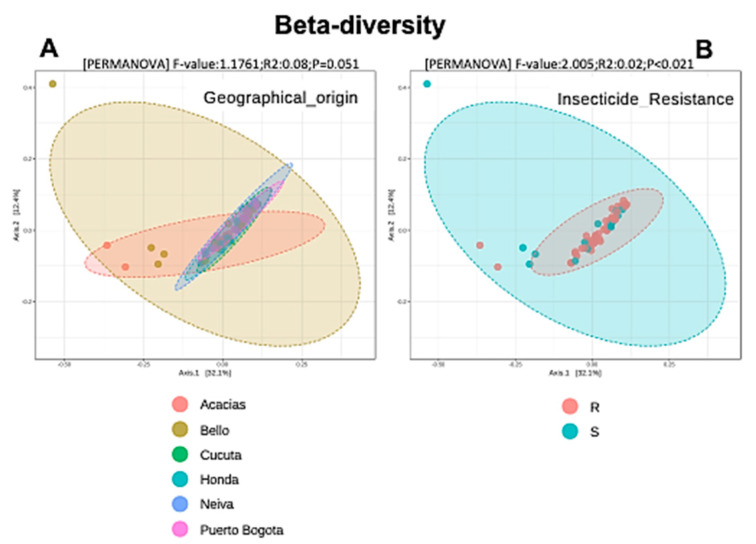
Principal coordinate analysis (PCoA) based on Bray-Curtis distances showing the diversity among six Colombian *Aedes aegypti* populations. (**A**) Beta diversity between geographical origins. (**B**) Beta diversity between insecticide-resistant and -susceptible mosquitoes. The observed diversity was only statistically significant among mosquitoes with different phenotypic profiles to lambda-cyhalothrin (*p* < 0.021).

The beta diversity analysis showed that the first two axes of the PCoA based on the Bray-Curtis distances yielded sample clusters that were consistent with our observed insecticide susceptibility patterns; the insecticide-resistant and -susceptible mosquitoes formed two distinct clusters (Figure 5B). These differences were statistically significant (R2 = 0.02, *p* < 0.021). In contrast, there were no significant differences due to geographical origin (PERMANOVA R2 = 0.08, *p*-value = 0.051, Figure 5A). These results were also supported by Jaccard and Jensen-Shannon indices (data not shown).

### 3.6. Taxonomic Composition and Abundance Analyses

**Figure 6:**
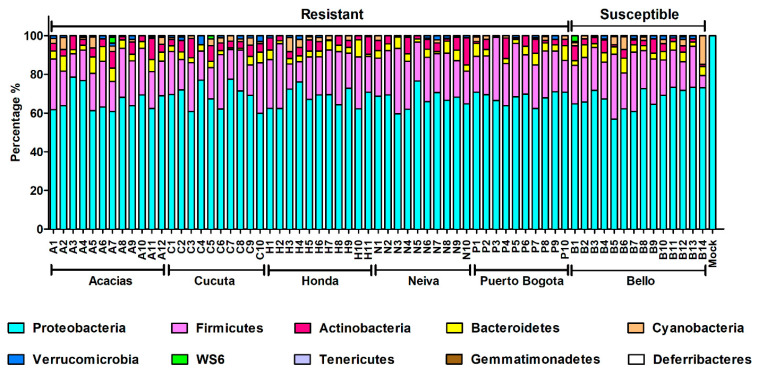
Comparison of the abundance of bacteria analyzed at the phylum level between lambda-cyhalothrin-resistant and -susceptible mosquitoes from six Colombian cities.

A core microbiome containing four main phyla (Proteobacteria, Firmicutes, Actinobacteria, and Bacteroidetes) was identified in all mosquitoes (Figure 6). The abundance of the phyla Cyanobacteria, Verrucomicrobia, WS6, Tenericutes, Gemmatimonadetes, and Deferribacteres was less than 4% in all mosquito populations studied (Figure 6); eight main classes (Alphaproteobacteria, Bacterioidia, Clostridia, Actinobacteria, Gammaproteobacteria, Erysipelotrichi, Bacilli, and Verrucomicrobiae); and 27 orders (the most abundant were Rickettsiales, Bacteroidales, Clostridiales, Actinomycetales, Rhodospirillales, Erysipelotrichales, Bacillales, and Verrucomicrobiales) were found. A heatmap of the most abundant orders identified in the midgut of mosquitoes from different cities and different resistance profiles is shown in Figure S4.

**Figure 7:**
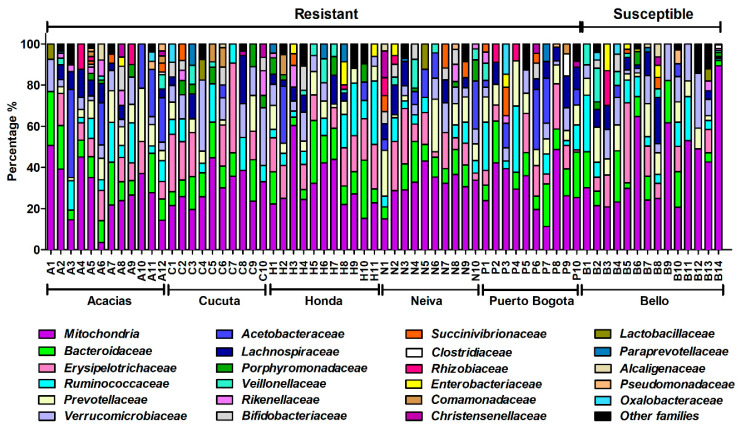
Comparison of the abundance of bacteria analyzed at the family level between lambda-cyhalothrin-resistant and -susceptible mosquitoes from six Colombian cities.

We found 36 families, where the 10 most abundant were: Mitochondria, Bacteroidaceae, Erysipelotrichaceae, Ruminococcaceae, Prevotellaceae, Verrucomicrobiaceae, Acetobacteraceae, Lachnospiraceae, Porphyromonadaceae, and Veillonellaceae (Figure 7).

We observed inter-individual variation in bacterial communities from the same geographic area. For example, a single-family (Mitochondria) comprises 51% of midgut bacteria in individual A1 from Acacias and is almost absent (3.5%) in another mosquito (A6) from the same population. Another example is found in the Bello population, where almost 90% of bacteria belong to the Mitochondria family in individual B14 and just 21% in B10. The same trend was observed for other families (Figure 7).

### 3.7. The Abundance Analyses Revealed Significant Differences Between Bacteria from Lambda-Cyhalothrin-Resistant and -Susceptible Mosquitoes

**Figure 8:**
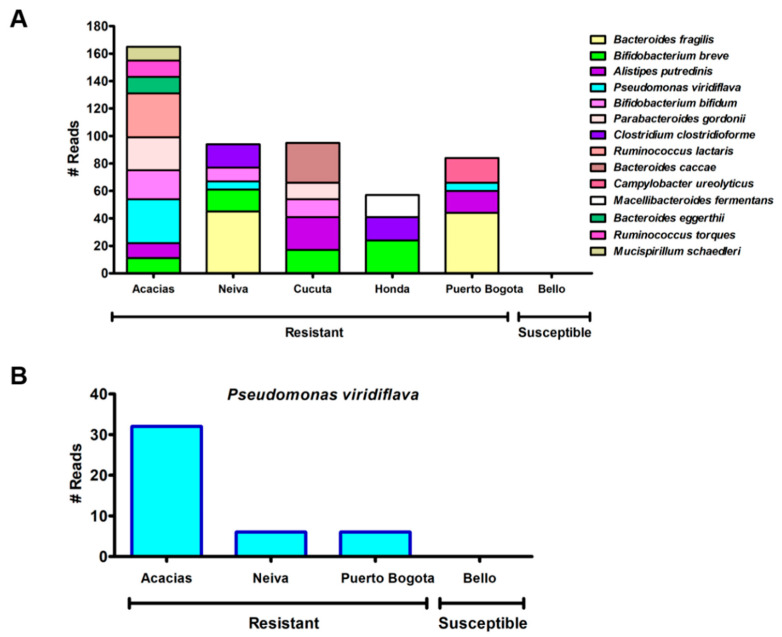
The number of reads for species present only in lambda-cyhalothrin resistant populations from Colombia. (**A**) Comparison of the number of reads per species present only in lambda-cyhalothrin resistant mosquitoes from six localities. (**B**) The number of reads for *Pseudomonas viridiflava* present only in Acacias, Neiva, and Puerto Bogota.

The composition and relative abundance of bacterial communities from clusters obtained previously were analyzed using the MetagenomeSeq statistical pipeline at class, order, family, genus, species, and OTU levels. We detected OTUs of bacteria that were only present in resistant (Table S6), or susceptible (Table S7) *Ae. aegypti.* The specific and common taxonomic levels for each phenotype are shown in Tables S8–S13. Interestingly, 14 species from the genera *Alistipes*, *Bacteroides*, *Bifidobacterium*, *Campylobacter*, *Clostridium*, *Macellibacteroides*, *Mucispirillum*, *Parabacteroides*, *Pseudomonas*, and *Ruminococcus* were found exclusively in resistant mosquitoes (Figure 8A, Table S13). We want to highlight the presence of *Pseudomonas viridiflava* that is associated with type II pyrethroid degradation [16,38]. This bacterium was exclusively present in the resistant populations of Acacias, Neiva, and Puerto Bogota (Figure 8B).

Biomarker analyses using LEfSe revealed 17 discriminant features with linear discriminant analysis (LDA) score >2 in susceptible mosquitoes, and only two features (OTUs 00008 and 00016) in resistant mosquitoes (Figure S5). A more detailed analysis of these results showed that the orders Rickettsiales and Clostridiales were statistically more abundant (LDA score >2, *p* < 0.05) in susceptible *Ae. aegypti* populations, while the orders Actinomycetales, Rhodospirillales, Erysipelotrichales, and Bacillales are significantly enriched (LDA score >2, *p* < 0.05) in resistant populations (Figure S6). We observed that the *Parabacteroides*, *Megasphaera*, *Akkermansia*, *Lardizabala*, *Ruminococcus*, and *Coprococcus* genera were enriched in susceptible mosquitoes (LDA score >2, *p* < 0.05) (Figure S7). Additionally, the genera *Clostridium* and *Oscillospira* were more abundant in resistant mosquitoes (LDA score >2, *p* < 0.05) (Figure S7). The species *Clostridium ramosum* was more abundant in resistant mosquitoes, while *Akkermansia muciniphila*, *Lardizabala biternata*, and *Coprococcus eutactus* were more abundant in susceptible mosquitoes (LDA score >2, *p* < 0.05) (Figure S8). At the genus and species level, the presence of *Pseudomonas viridiflava* was associated with resistant mosquitoes (Figure 8B, Figures S7 and S8). These results suggest that the gut microbiota may be altered by the resistant phenotype in *Ae. aegypti.*

## 4. Discussion

### 4.1. Evidence of High Resistance Ratio (RR) to Lambda-Cyhalothrin in Colombian Populations of Ae. aegypti

Many disparate Colombian mosquito populations have developed resistance to pyrethroid insecticides, especially lambda-cyhalothrin [5,6,7,8,9,10,20,23], despite their relatively short history of application compared with organophosphates and carbamates [9]. Maestre-Serrano et al. [23] evaluated the RR for pyrethroids such as deltamethrin, cyfluthrin, permethrin, and lambda-cyhalothrin in nine mosquito populations from the Colombian Caribbean region and found the highest levels corresponded to the use of lambda-cyhalothrin (RR > 10-fold; ranged from 4.9- to 83.3-fold) in seven of the nine populations [23]. Likewise, resistance to lambda-cyhalothrin has been found in Medellin, Giron, and Yumbo [10]; and other studies have documented a high RR in Cucuta (RR = 24.22) [39], Villavicencio (RR = 11.34) and Riohacha (RR = 10.96) [8]. In our results, four of the six populations evaluated had high levels of resistance (RR > 10), with the population of Cucuta (RR = 21.09) and Acacias (RR = 31.64) showing the highest levels.

### 4.2. Lambda-Cyhalothrin Resistance is Partially Explained by the 1016I Mutation

The pyrethroid resistance process may be associated with cross-resistance to DDT [5,10,40] and it is associated with *kdr* mutations, especially the 1016I mutation [8,10]. Although DDT has not been applied in Colombia since 1970, studies have shown that resistance to this insecticide has persisted in *Ae. aegypti* populations in Colombia and other regions of Latin America [5,20,40] after reinfestation by populations with genetically fixed resistance to DDT [5,6]. We had an allelic frequency range for this mutation between 0.04 and 0.59, which was comparable to other pyrethroid resistance studies from Colombia [8,9,10,23]. The 1016I mutation has been found in evaluated populations from Colombia, where a higher frequency of the mutation correlates with a higher level of resistance to lambda-cyhalothrin [8,10,23]. However, no directly proportional relationship between the degree of resistance and the 1016I mutation was found in another study [9]. These data support our findings, suggesting that other mechanisms could contribute to the development of lambda-cyhalothrin resistance in mosquito populations [9].

### 4.3. Midgut-Associated Bacteria Participate in the Resistance to Lambda-Cyhalothrin in Ae. aegypti

Aseptic rearing of larvae and treatment with antibiotics in adult stage reverted the highly lambda-cyhalothrin resistant phenotype of the Acacias population (Figure 3), supporting our hypothesis that the microbiome participates actively in the resistance to lambda-cyhalothrin.

A plethora of work has shown that insecticide resistance in *Ae. aegypti* is related to overexpression or gene amplification of enzyme families/classes, such as carboxy/choline esterases (CCEs), glutathione S-transferases (GSTs), cytochrome P450s (CYP450s), and mixed-function oxidases (MFOs) [5,6,7,10,41,42]. However, there are factors beyond these features that contribute to the resistant/susceptible phenotype. These include differential gene expression, copy number variation (CNVs), Single Nucleotide Polymorphisms (SNPs), epigenetic marks, and more recently, the microbiota [43,44,45,46,47,48].

Recently, an association was established between *Ae. aegypti* midgut bacteria and esterases and CYP450 activities [48]; there are midgut bacterial communities in *Ae. aegypti* associated with the detoxification metabolism of insecticides such as the carbamate propoxur and the organophosphate naled [48]. The elimination of bacteria in larvae with antibiotic treatment reduced esterases and CYP450 activities, consequently decreasing the metabolic detoxification of propoxur and naled [48]. It is not known exactly which bacterial communities are associated with these phenotypes but these results highlight the importance of the microbiota in the metabolic detoxification of carbamates and organophosphates. For this reason, we carried out a study of the diversity of bacteria associated with the midgut of *Ae. aegypti* populations with different degrees of resistance to lambda-cyhalothrin.

### 4.4. Aedes aegypti Midgut from Colombia Presented a Core Microbiota, Inter-Individual Variation, and Low Bacterial Diversity

There is no evidence of a relationship between *Ae. aegypti* microbiota and pyrethroid resistance, and much less in field populations. This study describes the first characterization of midgut microbiota from field derived Colombian mosquitoes with different resistance profiles to the insecticide lambda-cyhalothrin collected from diverse geographical locations. We compared the richness, diversity, composition, and relative abundance of midgut bacteria within these mosquito populations.

It has been described that a group of phyla formed by Proteobacteria, Firmicutes, Actinobacteria, and Bacteroidetes constitutes more than 99% of the total microbiota community in adult mosquitoes [49]. In our study, this group represented more than 96% of the total bacteria. These common bacteria constitute the “core microbiota” in adult mosquitoes because they have been consistently found [49,50,51,52,53], especially in the midgut of *Ae. aegypti* [33,54,55,56]. Within the remaining 4%, we found the candidate division WS6 phylum (Candidatus Dojkabacteria), which has not been found in previous studies. Similarly, another study has found rare phyla such as TM7 in *Ae. aegypti* [52]. Proteobacteria was the most abundant phylum, which was consistent with other studies [53,55,56,57].

Overall, the sequencing data showed that the structure and diversity of the midgut microbiomes are quite similar among the mosquito populations collected. This result is not surprising, as other studies have found that microbiota of *Ae. aegypti* from different geographic locations [53,58] or strains [54,59] were highly similar. This has also been shown in other species; there was a similar composition in the bacterial communities among adults of *Anopheles coluzzii* and *An. gambiae* [60], and between larvae of *Ae. aegypti* and *An. gambiae* [61]. Some authors have proposed that the similarity in bacterial communities may be due to the conditions under which mosquitoes are reared (laboratory colonization or field-collected), suggesting that environmental or host factors could shape the microbial community structure of mosquitoes [49,58,61,62]. However, similarity at the phylum, class, or family levels has been observed independent of environmental conditions or host factors [53,63,64]; therefore, the reason for the similarity in bacterial communities among *Ae. aegypti* populations is not yet clear [49].

The bacterial diversity of our populations does not seem to differ significantly based on geographical origin, temperature, climatic factors, or elevation. Studies with *Ae. aegypti* adults and larvae demonstrated that bacterial diversity was not affected by geographic area and larval habitat characteristics such as water temperature and pH, in agreement with our observations [53,64]. It is possible that other factors, including microbial interactions, mosquito genotype, and amino acid metabolic pathways, could shape mosquito microbiome communities [49,53,59,63].

Although similarity at the population level is commonly found in the mosquito microbiota, several studies in *Ae. aegypti, Ae. albopictus, An. gambiae, An. coluzzii, Culex quinquefasciatus*, and *Mansonia uniformis* have described high inter-individual variation [53,59,60,62,64], where certain bacteria members are prevalent in one individual but are rare or absent from others; this is found especially at lower classification levels [49,62,64], and it was observed at the family level in our study (Figure 7). It has been suggested that this condition may be important for metabolite production [65], or vector competence for the transmission of pathogens [62]. The role of inter-individual variability in mosquitoes is not well understood, but it could have a potential effect in resistance of field populations where insecticide pressure could shape bacterial communities, as seen in the *Riptortus–Burkholderia* symbiotic system, where the abundance of fenitrothion-degrading bacteria increased with the spraying of the pesticide [19,66].

In support of the previous idea, although there is a “core” midgut bacterial community composition in *Ae. aegypti*, there can be specific differences in some taxa [53,59]. We found populations of bacteria in low abundance that differ between lambda-cyhalothrin susceptible and resistant populations, which may play a role in metabolic processes of the mosquito associated with insecticide resistance. Previous studies have reported that the bacterial diversity (which reflects both the number and abundance of OTUs) in mosquito gut microbiota is low (<200 OTUs), compared with vertebrates [51], which is consistent with our findings (Table S4, Figure S4). The levels of diversity identified in this study are broadly similar to those of other holometabolous insects [51,61,62,64,67]. In fact, adult mosquitoes contain low diversity of bacterial communities [53,62,64]. These strong similarities do not negate the possibility that low abundance microbes, or their metabolites, could contribute to insecticide resistance.

### 4.5. Bacterial Taxa Associated with Lambda-Cyhalothrin-Resistant and -Susceptible Ae. aegypti Populations

Interestingly, we found that the gut microbiome reveals different relative abundances of groups of bacteria between lambda-cyhalothrin resistant and susceptible populations. At the species level, our analysis revealed the presence of *Pseudomonas viridiflava* in resistant populations from Acacias, Neiva, and Puerto Bogota, but not in the susceptible population from Bello. The genus *Pseudomonas* has been found previously in low frequencies in the mosquito midgut [64]. This bacterium is involved in the efficient degradation of fenvalerate, a type II pyrethroid principally used in agriculture, but also used in homes and gardens for insect control [16,38].

*Clostridium* was a common genus in susceptible and resistant mosquitoes but increased significantly in the latter (Figure S7). The abundance of *Clostridium ramosum* was significantly increased in all resistant populations, and *Clostridium clostridioforme* was unique to Neiva and Honda. *Clostridium* is associated with the degradation of fenpropathrin [68,69,70], a relatively new synthetic pyrethroid for controlling insect pests in agriculture and households, which has not been classified in the traditional pyrethroid classifications [71]. Another important genus was *Rhizobium*, which is related to the degradation of the insecticides malathion, an organophosphate [72], and imidacloprid, a neonicotinoid compound with high activity against a wide range of pests [73]. This genus was associated with resistant populations (Acacias, Neiva, and Puerto Bogota) in our study (Figure S7), with the presence of the species *Rhizobium daejeonense* (Figure S8). These results are novel for *Ae. aegypti* populations.

Some studies support the idea that the *Aedes spp.* microbiota and the human intestinal microbiome share bacteria because the mosquito is highly anthropophilic and shares a habitat with humans, breeding mostly in domestic water containers in and around dwellings [52,74]. In support of this, studies have verified that *Ae. aegypti* breeds significantly more in containers contaminated with *Escherichia coli* in field conditions [52,74]. Thus, some species found in the *Aedes spp.* microbiota, such as *E. coli* [52,74] and *Blautia* [75], are present in the microbiome of the human intestine [76,77]. In our study, we found several species that have also been associated with the human gut microbiota, such as *Blautia, Akkermansia muciniphila*, and *Oxalobacter formigenes* [76,77,78,79]. *Blautia* was associated with the resistant populations of Acacias, Cucuta, and Neiva. *Akkermansia muciniphila* and *Oxalobacter formigenes* were increased in the susceptible population of Bello.

Microorganisms play a significant role in degrading and detoxifying pyrethroids [69]. Many pyrethroid-degrading bacteria have been isolated and characterized: *Micrococcus sp.*, *Streptomyces aureus*, *Bacillus subtilis*, *Pseudomonas aeruginosa*, *P. stutzeri*, *Serratia sp.*, *Catellibacterium sp.*, and *Enterobacter asuburiae*, which biodegrade cypermethrin [16,69,70,80,81,82]; *Klebsiella sp.*, *Pseudomonas oleovorans*, *P. stutzeri*, and *Bacillus thuringiensis* which participate in the biodegradation of lambda-cyhalothrin [11,16,66,69]; *Brevibacterium aureum*, *Catellibacterium sp.*, *P. aeruginosa*, *Serratia marcescens*, *Sphingobium sp.*, *B. thuringiensis*, and *Arthrobacter nicotinovorans* which degraded deltamethrin [11,69,70]; *Bacillus cereus*, *Stenotrophomonas sp.* and *Pseudomonas viridiflava*, reported in the present study, which have a role in the degradation of fenvalerate [16,38,69,70].

In our study, bacteria from the orders Actinomycetales, Rhodospirillales, Erysipelotrichales, and Bacillales are significantly enriched in resistant populations. Likewise, Vijayakumar et al. [83] reported that bacteria belonging to the orders Bacteroidales, Enterobacteriales, Clostridiales, Burkholderiales, Lactobacillales, and Bacillales were significantly more abundant in pesticide-resistant populations of the brown planthopper *Nilaparvata lugens*, one of the most important pests of rice [83]. In *Plutella xylostella*, a destructive pest of cruciferous crops, it was found that the order Lactobacillales (more abundant) and much scarcer taxa such as Pseudomonadales and Xanthomonadales were associated with the midgut and conferred resistance to chlorpyrifos and fipronil [15]. This was similar to our study, where *Pseudomonas viridiflava*, a scarce taxon belonging to the order Pseudomonadales, was associated with insecticide-resistant populations.

The bacterial load of a particular species may influence insecticide resistance [18]. This load, in turn, could be determined by selection pressures [12,18] or the presence of resistance mutations [18]. A study in mosquitoes has demonstrated differing composition of the microbiota and its function between fenitrothion-susceptible and resistant strains of *An. albimanus*. Lower bacterial diversity and significant enrichment of organophosphate-degrading bacteria were observed in the resistant population, suggesting the enrichment of bacterial taxa with a competitive advantage in response to insecticide selection pressure [12]. This is in line with a study that demonstrated that in resistant populations of *Culex pipiens*, the load of *Wolbachia* was higher in resistant mosquito strains and that this can change with selection pressure. The presence of resistance alleles was directly related to the presence of the bacteria [84]. This is striking because in our study the Acacias population, which had the highest RR and a high allelic frequency for the 1016I mutation, had the highest number of reads for *Pseudomonas viridiflava, Clostridium ramosum*, and *Rhizobium daejeonense*. Although more experiments are needed, these data suggest that the composition of the microbiota can change in response to a resistance mutation such as 1016I. These data are correlative and there is no evidence yet that specific bacteria cause the resistance.

Finally, future studies should investigate the role of microbiota in GST, CCE, and CYP450 metabolic mechanisms in populations of insecticide-resistant *Ae. aegypti* from Colombia and around the world. Our new challenge is to understand and elaborate on the mechanisms used by these midgut-associated bacteria to metabolize insecticides such as lambda-cyhalothrin. However, whether the microbiome influences the resistance or the resistance influences the microbiome should be studied in more detail in future studies. Additionally, experiments introducing bacteria associated with the resistant phenotype into axenic susceptible populations are needed to evaluate the type and number of symbionts that are required to change their resistance profile.

## 5. Conclusions

This survey provides new data regarding the microbial composition and abundance between *Ae. aegypti* populations with different insecticide resistance profiles, which may reflect different functional requirements in both groups of mosquitoes. A comprehensive knowledge of the microbiome present in the midguts of insecticide-resistant mosquitoes is fundamental to better understand what role bacteria, such as *Pseudomonas viridiflava*, *Clostridium ramosum, C. clostridioforme*, and *Rhizobium daejeonense*, associated with these populations may play in mediating resistance. Our microbiome data provide an essential baseline for future vector control intervention studies, which should focus on bacteria implicated in the detoxification processes in field populations.

## Figures and Tables

**Table 1:** Insecticide phenotypic profile (resistance ratio: RR) of *Aedes aegypti* populations collected in six Colombian cities against lambda-cyhalothrin.

Cities	RR_50_	Phenotype
Bello (B)	1.97	Susceptible
Puerto Bogota (P)	9.14	Moderate Resistant
Neiva (N)	10.55	High Resistant
Honda (H)	11.81	High Resistant
Cucuta (C)	21.09	High Resistant
Acacias (A)	31.64	High Resistant

## Supplementary Materials

The following are available online at https://www.mdpi.com/2075-4450/11/9/584/s1, Figure S1: The number of reads obtained in individual mosquito from different Colombian cities, Figure S2: Rarefaction curves of individual mosquito libraries of *Ae. aegypti* from different Colombia cities, Figure S3: Rarefaction curves of individual mosquito libraries of *Ae. aegypti* from different phenotypic profiles to insecticide, Figure S4: Heatmap of identified order in mosquito midguts from different cities and resistance profiles to insecticides, Figure S5: LEfSe analysis of OTUs differentially abundant between resistant (R) and susceptible (S) mosquitoes, Figure S6: LEfSe analysis of orders differentially abundant between resistant (R) and susceptible (S) mosquitoes, Figure S7: LEfSe analysis of genera differentially abundant between resistant (R) and susceptible (S) mosquitoes, Figure S8: LEfSe analysis of species differentially abundant between resistant (R) and susceptible (S) mosquitoes, TableS1_ Metadata file: Geographical origin, phenotypic profile to insecticide, and code of each mosquito analyzed. TableS2_OTU_Complete data: OTUs identified and the number of reads in 68 samples of *Ae. aegypti* and mock microbiota. TableS3_OTU with Phylum: OTUs annotated by phylum and number of reads in 68 samples of *Ae. aegypti* and mock microbiota. TableS4_Observed OTUs: Number of OTUs observed in 68 DNA samples (67 from mosquito midguts of *Ae. aegypti*, and the mock microbiota). TableS5_Filter: Total number of reads by each city with the annotated OTUs of *Ae. aegypti* and mock microbiota. Each city is represented with a different color to filter the OTUs found specifically in each locality. TableS6_Only Resistance: OTUs exclusively identified in the *Ae. aegypti* resistant populations to lambda-cyhalothrin. TableS7_Only Susceptible: OTUs exclusively identified in the *Ae. aegypti* susceptible population to lambda-cyhalothrin. TableS8_Phylum: Phyla exclusively identified in *Ae. aegypti* resistant and susceptible populations to lambda-cyhalothrin and common between both phenotypes (red). TableS9_Class: Classes exclusively identified in *Ae. aegypti* resistant and susceptible populations to lambda-cyhalothrin and common between both phenotypes (red). TableS10_Order: Orders exclusively identified in *Ae. aegypti* resistant and susceptible populations to lambda-cyhalothrin and common between both phenotypes (red). TableS11_Family: Families exclusively identified in *Ae. aegypti* resistant populations to lambda-cyhalothrin and common between both phenotypes (red). TableS12_Genus: Genera exclusively identified in *Ae. aegypti* resistant and susceptible populations to lambda-cyhalothrin and common between both phenotypes (red). TableS13_Species: Species exclusively identified in *Ae. aegypti* resistant and susceptible populations to lambda-cyhalothrin and common between both phenotypes (red).
