# Supplementary material for: The Midgut Microbiota of Colombian Aedes aegypti Populations with Different Levels of Resistance to the Insecticide Lambda-cyhalothrin
Source: Insects. 2020 Sep 1;11(9):584. doi: 10.3390/insects11090584 (PMC7565445; doi:10.3390/insects11090584)
Supplement: Supplementary file 1 [file insects-11-00584-s001.zip › insects-883458-Supplementary Material Figure S1-S8.docx]

The midgut microbiota of Colombian *Aedes aegypti* populations with different levels of resistance to the insecticide lambda-cyhalothrin

Supplementary Material

**Supplementary Figure S1.** The number of reads obtained in individual mosquito from different Colombian cities. The code and geographical origin are specified in the supplementary excel tables file: TableS1.

**Supplementary Figure S2.** Rarefaction curves of individual mosquito libraries of *Ae. aegypti* from different Colombia cities.

**Supplementary Figure S3.** Rarefaction curves of individual mosquito libraries of *Ae. aegypti* from different phenotypic profiles to insecticide.

**Supplementary Figure S4.** Heatmap of identified order in mosquito midguts from different cities and resistance profiles to insecticides.

**Supplementary Figure S5.** LEfSe analysis of OTUs differentially abundant between resistant (R) and susceptible (S) mosquitoes. LDA score=2 and *p*<0.05 were considered significant.

**Supplementary Figure S6.** LEfSe analysis of orders differentially abundant between resistant (R) and susceptible (S) mosquitoes. LDA score=2 and *p*<0.05 were considered significant.

**Supplementary Figure S7.** LEfSe analysis of genera differentially abundant between resistant (R) and susceptible (S) mosquitoes. LDA score=2 and *p*<0.05 were considered significant.

**Supplementary Figure S8.** LEfSe analysis of species differentially abundant between resistant (R) and susceptible (S) mosquitoes. LDA score=2 and *p*<0.05 were considered significant.
